# Long-term durability of discarded cork-based composites obtained by geopolymerization

**DOI:** 10.1007/s11356-024-33958-8

**Published:** 2024-06-12

**Authors:** Giovanni Dal Poggetto, Luisa Barbieri, Antonio D’Angelo, Alfonso Zambon, Paolo Zardi, Cristina Leonelli

**Affiliations:** 1 Department of Engineering “Enzo Ferrari”, University of Modena and Reggio Emilia, Via P. Vivarelli 10, 41125 Modena, Italy; 2 Department of Engineering, University of Campania “Luigi Vanvitelli”, Via Roma 29, 81031 Aversa, Italy; 3 Department of Chemical and Geological Sciences, University of Modena and Reggio Emilia, Via G. Campi 103, 41125 Modena, Italy

**Keywords:** Discarded cork, Cork-based composites, Geopolymerization, Durability, Chemical properties, Microstructural properties, Antibacterial properties, Physical properties

## Abstract

Geopolymers are amorphous aluminosilicate inorganic polymers synthesized by alkaline activation characterized by a lower carbon footprint, greater durability, and excellent mechanical properties compared to traditional concrete, making them promising building materials for sustainable construction. To develop sustainable lightweight geopolymer-based building materials useful as fire resistant thermal insulation materials, we added 5 and 10 wt% of discarded cork dust, a readily available industrial by-product, to metakaolin before and after the alkaline activation with sodium hydroxide 8 M and sodium silicate solutions. We followed the chemical, microstructural, antibacterial, and physical properties of the resulting composites for up to 90 days in order to monitor their long-term durability. The presence of cork does not interfere with the geopolymerization process and in fact reduces the density of the composites to values around 2.5 g/cm^3^, especially when added after alkaline activation. The composites resulted in chemically stable matrices (less than 10 ppm of cations release) and filler (no hazardous compounds released) with a bacterial viability of around 80%. This study provides valuable insights into the tailoring of discarded cork-based composites obtained by geopolymerization with a porosity between 32 and 48% and a mechanical resistance to compression from 15 to 5 MPa, respectively, suggesting their potential as durable interior panels with low environmental impact and desirable performance.

## Introduction

Sustainable chemistry and engineering aim to the design of products and processes that minimize or eliminate the use and generation of hazardous substances, optimize the use of matter and energy throughout their life cycle, exploit residues as secondary raw materials, minimize human exposure to potential hazards (including reducing toxicity), and reduce pollution. Construction materials can be divided into two macrofamilies: heavy ones, such as concrete and steel, and light ones, such as aerated concrete, foam concrete, lightweight aggregate concrete, and lightweight steel. The former has significant limitations: they are usually heavy, energy-intensive to produce, and cumbersome to transport. Conversely, lightweight materials can reduce the overall weight of a structure and the associated material and transportation costs, increase energy efficiency, and minimize both carbon emissions during production and the demand for non-renewable resources. Regarding cement, the most widely used construction material in the world, the evolution of the standards towards more sustainable materials has taken into account two aspects: (i) the reduction of CO_2_ emissions and (ii) the increase in the use of recycled materials. Two new European standards, already implemented in Italy, go in this direction. EN 197–5: 2021 (EN 197–5: 2021) deals with the first aspect. It standardizes new compositions of ternary cements (i.e., containing two main constituents in addition to Portland clinker) with a lower percentage of clinker. Regarding the latter aspect, EN 197–6: 2023 (EN 197–6: 2023) specifies cement with recycled concrete fines whose intended use is the production of concrete, mortar, grout, etc., in order to support circularity. However, ordinary concrete and cement have disadvantages, such as low thermal and fire resistance, poor chemical resistance to acids and salts, and significant global carbon dioxide emissions (Bakhtyar et al. 2017); therefore, there is a strong impulse to research more efficient and ecological building materials. In this context, geopolymers appear to be promising candidates, especially as a replacement for conventional Portland cement (Davidovits and Davidovics 1988, 1998; Duxson et al. 2007). Geopolymers are amorphous aluminosilicate inorganic polymers with 3D frameworks consisting of tetrahedral silicate and aluminate units joined through their shared oxygen atoms. The negative charges on the alumina tetrahedra are compensated for by extra-framework monovalent alkali cations, usually Na^+^ or K^+^.

Geopolymers are synthesized via alkaline activation of aluminosilicate materials and present remarkable mechanical (high strength), physical (permeability), thermal (temperature resistance), and chemical (acid resistance) properties. The first three properties are related to one of the most dominant characteristics of geopolymers, i.e., the porous structure (Chen et al. 2019; Zhang et al. 2010; Ma et al. 2013) which develops on different size scales and is introduced into the material framework without the need for costly structural directing agents. The porosity percentage range (measured by mercury intrusion porosimetry) of the traditional metakaolin-based geopolymer is 34–44 (Chen et al. 2021), and it is higher than that of ordinary Portland cement, for which a water/cement ratio larger than 0.6 is required (Haga et al. 2005), which is not commonly used in construction. In addition, the micro pores in geopolymers are usually finer than in ordinary Portland cement (Provis et al. 2011; Rovnaník 2010), and it causes a correlation with the sorption and capillary condensation and also contributes to the thermal insulation of geopolymers. Geopolymers are ease to synthesize at room temperature starting from both naturally occurring precursors and industrial wastes, and offer the possibility of being functionalized thus tailored for a wide range of applications. Compared to traditional cement, geopolymers have a lower carbon footprint, greater durability (e.g., the performance of geopolymer made from metakaolin (MK) alone is promising, especially in aggressive situations such as sulphuric acid attack on sewer pipes where Portland cement concretes are vulnerable), and excellent mechanical properties, making them the ideal choice for sustainable construction (Davidovits and Davidovics 1998; Duxson et al. 2007; Chang et al. 2005). Progress has been made in the use of geopolymers in the construction sector, both in the treatment and restoration of cultural heritage and in the field of building, architecture, and construction in general. For the first aspect, the ability of traditional metakaolin-based geopolymer mortars to repair material by filling cracks (Geraldes et al. 2016; Moutinho et al. 2020; Clausi et al. 2016) and to consolidate old stone constructions has been demonstrated (Baltazar et al. 2019). There is literature on the use of silica fume to confer high strength (Bajpai et al. 2020), ground granulated blast slag to refine pores and improve resistance to alkali-silica and sulphate reactions (Dimas et al. 2009; Hadjsadok et al. 2012), rice husk ash for its important pozzolanic activity (Nair et al. 2008), red mud with high alkalinity and pH values of 10 to 13 (Ye et al. 2016), and glass powder together with other precursors in the production of geopolymers (Dal Poggetto et al. 2021a). In analogy with other classes of building materials, therefore, there is a strong research drive towards the use of recycled materials as sources for the synthesis of geopolymers (Zaid et al. 2022; Sudagar et al. 2018; Novais et al. 2019), which can also functionalize the finished product. Among the various properties, the incorporation of lightweight waste materials, which facilitates the potential applications of geopolymers as possible insulation materials, has been little explored, as highlighted in existing research (Zaid et al. 2022; Samuel et al. 2023). Among the waste materials with lightweight properties, the waste from cork stoppers processing stands out for its properties, and there is little literature on its valorization as a lightweight filler or aggregate. Sudagar et al. and Novais et al. (Sudagar et al. 2018; Novais et al. 2019) reported that the incorporation of cork residue as a filler in a geopolymer matrix could reduce water consumption during production and extend the setting time, thereby prolonging the geopolymerization process. In addition, the advantageous properties of cork, such as its lightness, impermeability to liquids and gases, chemical resistance, resistance to biological corrosion, thermal insulation, acoustic and anti-vibration properties, incombustibility, elasticity, durability, health neutrality, and mechanical strength, could be exploited.

With this experimental study, the authors aim to contribute to the implementation of the range of waste materials used in the field of geopolymers, and using one that is well suited to a geopolymer matrix due to its properties such as porosity, lightness, and fire resistance. In this paper, we investigate the effect of adding 5 and 10 wt% of cork dust to geopolymer paste before and after the alkaline activation. We considered the sustainability of both the materials and the process, i.e., by using cork powder, an industrial by-product, as received, and by using Na-based reagents as activating solutions and room temperature curing of the resulting composites. We carried out chemical, microstructural, antibacterial, and physical studies on samples aged for 28, 60, and 90 days in order to assess the long-term durability of the discarded cork-based composites obtained.

## Experimental methods

### Materials

We used ARGICAL™ M1000 metakaolin (MK) supplied by Imerys in France. According to the manufacturer’s specifications, this MK consists of SiO_2_ (55 wt%), Al_2_O_3_ (40 wt%), Fe_2_O_3_ (1.4 wt%), TiO_2_ (1.5 wt%), Na_2_O + K_2_O (0.8 wt%), CaO + MgO (0.3 wt%), and LOI (1 wt%) (Dal Poggetto 2021a). At the same time, we used discarded cork (DC) derived from the production of agglomerated cork bottle caps by Italsughero, a local company in Montecchio Emilia (RE), Italy. This dust is generated specifically during the smoothing phase of agglomerated cork caps and is collected by a cyclonic air filtration system. The particles in this cork by-product range of diameter from 0.063 to 1 mm and may contain polyurethane adhesive and paraffin, which are used as binders and additives for cork particles, respectively. Further details on the characterization of the discarded cork can be found in a previous study by B. Malchiodi et al. (Malchiodi et al. 2022). For the preparation of the NaOH solution, we used laboratory grade granules (96 wt%, Sigma-Aldrich, Italy) dissolved in distilled water to obtain a concentration of 8 M. In addition, our formulations included a sodium silicate solution supplied by Ingessil, Verona, Italy. This sodium silicate solution had a SiO_2_/Na_2_O molar ratio of 3.00, a SiO_2_ content of 26.50 wt%, a Na_2_O content of 8.70 wt%, and a pH of 11.7. The solution had a bulk density of 1.34 at 20 °C.

### Preparation of geopolymer specimens

To formulate the geopolymeric binder to be used as a reference, designated as GP0, we adopted an optimized formulation (Dal Poggetto et al. 2021a) (Table 1), which combines a specific amount of dry metakaolin powder with an alkaline solution containing 8 M NaOH and soluble Na-silicate, using continuous mechanical stirring. We then incorporated two amounts (5% and 10% by weight) of discarded cork dust both before and after the alkali activation. This process resulted in different geopolymer composites named as GP-5DC and GP-10DC (before alkali activation), and 95GP0-5DC and 90GP0-10DC (after alkali activation), as detailed in Table 1. In order to obtain a good metakaolin reticulation/geopolymerization, we kept the proportion of oxides constant for all the formulations, with a very low variability of the water content.

**Table 1:** Geopolymer formulations expressed in grams per each raw material and in main oxides (wt%). L/S = liquid to solid ratio, where liquid is the sum of Na-silicate and NaOH solutions and solid is the sum of MK and DC. H_2_O (wt%) is the sum of the water present in the two alkaline solutions

**Sample**	**MK (g)**	**Na-silicate sol. (g)**	**NaOH sol. (g)**	**Discarded cork (g)**	**L/S (wt/wt)**
GP0	100	40	38	0	0.78
GP-5DC	95	38	36.5	5	0.75
GP-10DC	90	36	34	10	0.70
95GP0-5DC	53	21	20	5	0.71
90GP0-10DC	51	20	19	10	0.64
**wt%**	**SiO**_**2 without cork/with cork**_	**Al**_**2**_**O**_**3 without cork/with cork**_	**Na**_**2**_**O **_**without cork/with cork**_	**Other oxides **_**without cork/with cork**_	**H**_**2**_**O **_**without cork/with cork**_
GP0	37	22	6	2	32/32
GP-5DC	37/36	22/22	6/6	2/2	32/31
GP-10DC	37/35	23/21	6/6	2/2	32/30
95GP0-5DC	37/35	23/21	6/6	2/2	31/30
90GP0-10DC	37/33	23/20	6/5	2/2	31/28

**Fig. 1:**
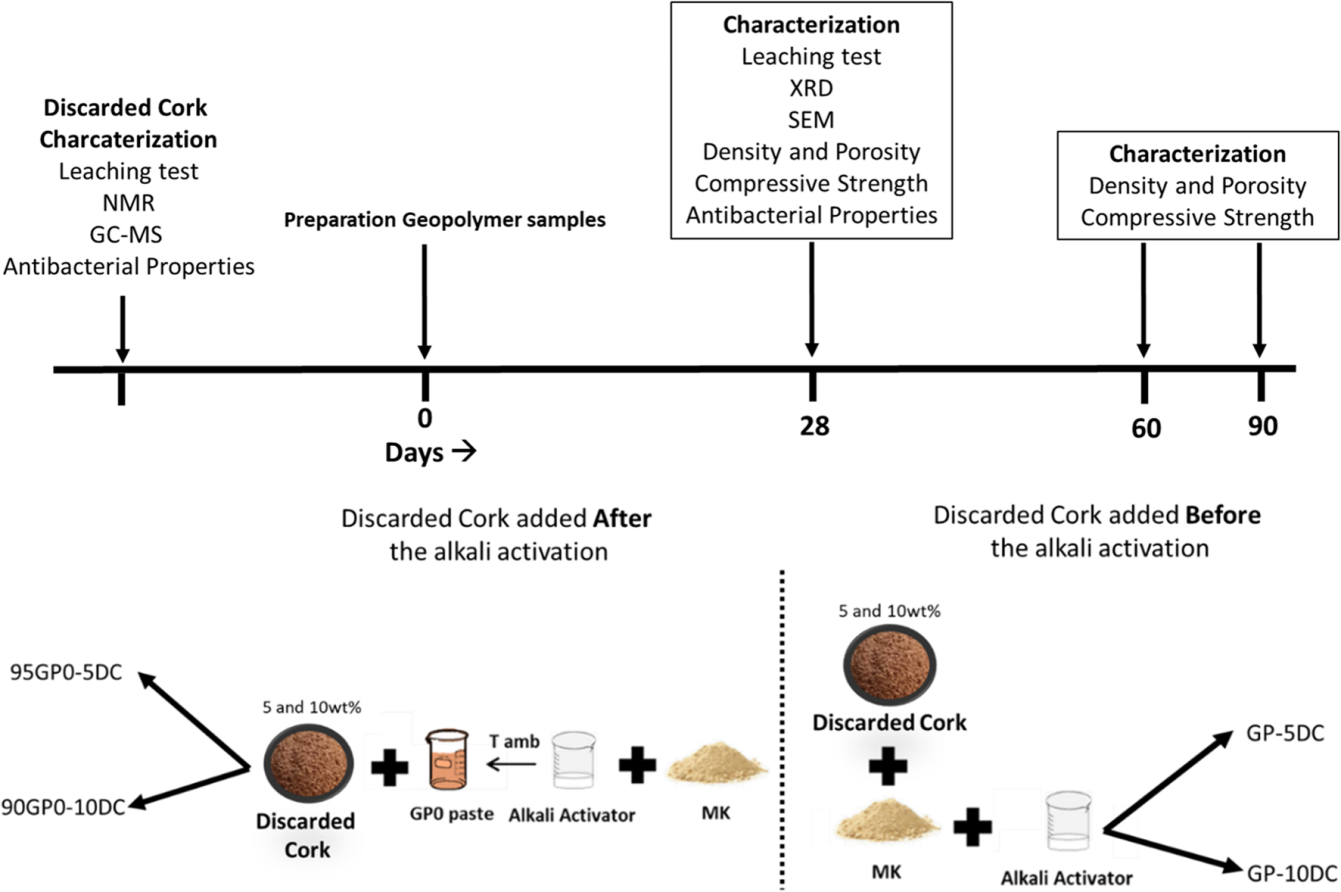
Flowchart of the process

The preparation of the fresh paste involved careful mixing of powders and liquids using a planetary mixer (Aucma 1400W, Aucma CO., LTD., Shandong, China). The freshly prepared pastes, which were of similar workability, were carefully poured into cubic molds with dimensions of 30 × 30 × 30 mm3. After ensuring that any trapped air bubbles were removed using a vibrating table, the molds were meticulously sealed. The geopolymers were allowed to cure at room temperature in an environment of 100% relative humidity. After 1 day of curing, the molds were opened, and the cured geopolymers were exposed to the laboratory atmosphere until characterization. A minimum of 12 samples were produced per each formulation, and full characterization was carried out on all samples after 28, 60, and 90 days of ageing. The flowchart of the methodology is reported Fig. 1.

### Leaching test

The assessment of the leaching potential of Al^3+^ and Si^4+^ ions due to incomplete reticulation, as well as the release of heavy metals from discarded cork powder, was carried out on all geopolymer formulations in accordance with the EN 12457–2:2004. After crushing and sieving the geopolymer to particles smaller than 3 mm, they were immersed in milliQ water at a solid-to-water weight ratio of 1:10 and left for 24 h. The leachate solutions, filtered to remove particles smaller than 0.45 µm, were then collected. These filtered solutions were then acidified to pH = 2 using HNO_3_ (69%) solution. Following the guidelines outlined in EN ISO 11885:2009, titled "Water Quality-Determination of selected elements by inductively coupled plasma optical emission spectrometry (ICP-OES)”, the concentrations of ionic heavy metals were determined using ICP-OES equipment from Agilent, Santa Clara, CA, USA. All ionic metals concentrations are expressed in parts per million (ppm). The limit of quantitation (LOQ) for Al, B, Ba, Fe, Mn, Sb, and Zn was set at 5 parts per billion (ppb). Meanwhile, the limit of detection (LOD) for Be, Cd, Co, Cr, Cu, Mo, Ni, Pb, Se, Sn, and V was set at 2 ppb. In addition, the LOQ for Si and Ca has been maintained at 500 ppb.

### Chemical and NMR analyses on eluate

Proton nuclear magnetic resonance (^1^H NMR) and gas chromatography-mass spectrometry (GC–MS) were used to identify possible low-molecular weight organic compounds released from geopolymers in the leachate solutions described above. For the NMR analysis, 5 mL of each leachate solution was first extracted in ethyl acetate (EtOAc, 3 × 5 mL) and dichloromethane (DCM, 3 × 5 mL), the organic phases were evaporated under reduced pressure, and the residues were dissolved in deuterated chloroform (CDCl_3_, 0.75 mL). A volume of 5 mL of the leachate solutions was then lyophilized and dissolved in esadeuterated dimethyl sulfoxide (DMSO-d6). ^1^H spectra (32 scans) were recorded at 298 K on Bruker FT-NMR Advance 400 (400.13 MHz) and Bruker FT-NMR Advance III HD 600 (600.13 MHz). Chemical shift values were recorded in ppm relative to TMS and were determined by taking as reference the isotopic impurity signals of CDCl_3_ (7.26 ppm for ^1^H) and DMSO-d6 (2.50 ppm for ^1^H). Data are given as follows: chemical shift (δ) in ppm, multiplicity, and coupling constants (J) given in Hertz.

For the GC–MS analysis, a volume of 10 µL of each extract was analyzed first, followed by 10 µL of the leachate solutions. GC–MS data were acquired on an Agilent 7890B system equipped with a J&W HP-5 ms GC column (30 m, 0.25 mm, 1.00 µm) and coupled to an Agilent HP 5973 MSD detector; mass was scanned in the range 35–400 Da.

### Mineralogical composition

A thorough analysis of the crystalline phases in the geopolymer formulation containing 5% cork dust cured for 28 days was carried out using the X-ray diffraction (XRD) technique. The XRD analysis was performed on an X’Pert PRO instrument from PANAlytical, Malvern Panalytical Ltd., Malvern, UK. The X-ray diffractometer was operated at 40 kV and 40 mA, using Cu-Kα radiation with Ni filtration. Diffraction patterns were collected in the 2θ range from 5 to 70° using the X’Celerator detector, with a step size of 0.02°, a counting time of 3 s, and a slit width of 10. To identify the mineral phases present, we compared the experimental diffraction peaks with reference patterns using the DIFFRAC plus EVA software (2005 PDF2) from Bruker, Billerica, MA, USA. To ensure the accuracy of our results and to avoid preferential orientation, a side charge was applied to the powdered sample during the analysis. This meticulous approach provided us a reliable insight into the composition and structure of the geopolymer formulation containing cork waste.

### Apparent density, real density, and porosity

Apparent density (*ρa*) was calculated by dividing the measured mass of the cubic specimens by their predetermined volume, providing a geometric determination of density. To ensure accuracy, three independent measurements were made for each solidified composite, and the average apparent density was calculated from these values. On the other hand, the real density (*ρr*) was determined using a helium pycnometer (Micrometrics Accupyc 1330, Micrometrics Instruments, Norcross, GA 30093, USA). The total porosity (*P*%) was calculated using the following formula from the real and apparent density values obtained:

1 $$P\%=\left(1-\rho a/\rho r\right)\cdot100$$

### Microstructural analysis

Environmental scanning electron microscopy (ESEM) using an ESEM-Quanta200-FEI instrument was chosen to examine the microstructure of the samples cured for 28 days. The primary objective of this analysis was to evaluate the development of the geopolymeric amorphous phase and to identify any unreacted MK particles within the samples. Secondly, the fracture surface was observed to detect the interface between the cork and the geopolymeric matrix and possible toughening mechanisms, and to check the pore distribution. To prepare the specimens for SEM analysis, a thin layer of gold was sputtered onto the freshly fractured surface of each specimen.

### Mechanical properties

After 28, 60, and 90 days of curing, the mechanical properties of the cubic specimens were evaluated by compression tests. These tests were conducted using a Controls L1052 Testing Machine (Cernusco (MI), Italy). The compressive strength values reported here are the average results from eight tests, exhibiting a minor variation of up to 2%.

### Antibacterial properties

The hardened samples were evaluated for their potential antibacterial activity against both Gram-negative (*Escherichia coli*, ATCC® 25922™) and positive (*Staphylococcus aureus*, ATCC® 25923™) microbial strains using the Kirby-Bauer method (Hudzicki 2009). The detailed procedure for preparation of bacterial media and strain dissolution can be found elsewhere (D’Angelo et al. 2023; Righi et al. 2022). Regarding sample preparation, all samples were carefully ground using a mortar and pestle, compressed to obtain discs of 1.30 ± 0.05 cm and sterilized under UV light for 1 h before their contact with the bacterial strains (as in accordance with the procedure reported in Catauro et al. (Catauro et al. 2023)). To perform the test, the *E. coli* and *S. aureus* bacterial suspensions (10^9^ CFU/mL) were plated on their specific solid media (i.e., *E. coli* on TBX medium (Tryptone Bile X-Gluc, Liofilchem, Italy) and *S. aureus* on Baird-Parker agar (Liofilchem, Italy)), and incubated for 24 h without (for the control) and with 100 mg of sample powders. In particular, *E. coli* was incubated at 44 °C, and *S. aureus* was incubated at 36 °C. At the end of the incubation period, the diameter of the inhibition zone and the reduction in bacterial viability (BV, %) were assessed. Three replicates were made for each sample to determine the average absolute deviation (AAD). The BV has been calculated in accordance with Catauro et al. (Catauro et al. 2021).

## Results and discussion

All samples containing cork were visually inspected for the absence of efflorescence after 28 days. The high SiO_2_/Na_2_O molar ratio and low Na_2_O content (Longhi et al. 2020) ensured the chemical stability of the cured product even though the cork dust added to the fresh paste partially adsorbed the activator solution. Immersion tests in water were carried out to better qualify and quantify the chemical stability of the geopolymerized matrix and cork filler.

### Leaching test

The data presented in Fig. 1 illustrate the amounts of the most abundant metals released as ions, i.e., aluminum (Al), calcium (Ca), iron (Fe), potassium (K), magnesium (Mg), sodium (Na), and silicon (Si). The remaining heavy metals measured (Ag, AS, B, Be, Cd, Co, Cr, Cu, Mo, Ni, Pb, Se, Sn, Tl, V, and Zn) are not reported as their concentrations were consistently below 0.01 parts per million (ppm). It should be noted that the amount of soluble species released is extremely low, less than 10 ppm for all the cations studied, indicating an extremely stable geopolymeric structure for all the formulations.

**Fig. 2:**
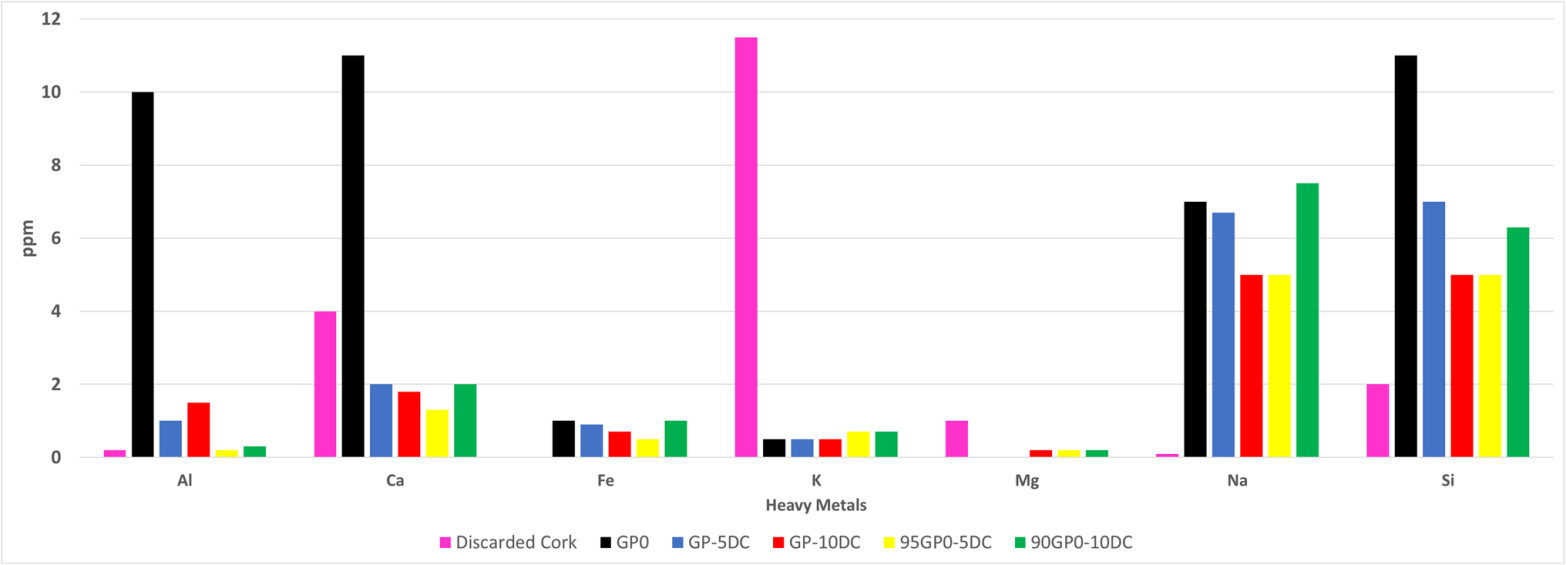
Results of the leaching test of the discarded cork and the geopolymers samples

Figure 2 clearly shows that aluminum is mainly released from the metakaolin-based geopolymer, while in the other samples, the release of Al remains below 2 ppm. Similarly, GP0 shows the most significant silicon release. The reduction in silicon release is halved when moving from GP0 to the geopolymer composites, either the series of DC added before or after the alkali activation. The formulations containing cork dust show a higher reticulation of silicon, since its release is lower with respect to GP0. For calcium release, GP0 shows the highest values, while for all the other geopolymers the calcium release decreases. Potassium release from the as-received cork dust is particularly high, in agreement with the results of previous studies (Malchiodi et al. 2022), but when the residue is incorporated into the geopolymer matrix, either before or after the alkaline activation phase, the value decreases significantly. Conversely, the presence of sodium remains relatively constant in the samples and in the reference sample GP0, and the contribution of DC is almost absent. This behavior is in agreement with that observed in the XRD spectra (see the dedicated paragraph) and indicates an unaffected geopolymerization reaction, since Na^+^ is bound to similar Al–O–Si– structures present in GP0. Looking more closely at the data for the geopolymers (Fig. 2), it can be observed that the variability in Na^+^ release is similar to that of the Si-bearing species. This trend can be attributed to the Na coming from the initial alkali activator (8 M NaOH and Na-silicate solution). Furthermore, although the presence of iron (Fe) and magnesium (Mg) is extremely low (below 2 ppm), they are still detectable in the eluates. In the case of Fe, it is absent in the DC. It is noteworthy that the release of Fe is only evident in the geopolymer samples, indicating that it originates from the original metakaolin. Conversely, for Mg, the situation is reversed; it is evident that the released Mg is absent in GP0 but present in cork and subsequent geopolymers, except for GP-5DC, probably due to the low percentage of DC introduced in the metakaolin before alkaline activation.

As a general comment, we can state that the low amount of ions released in water indicates a very efficient reticulation/geopolymerization of the hardened samples, in agreement with published data (Adhikary et al. 2024; D’Angelo et al. 2022; Boldrini et al. 2021). As a reference for the reticulation of the geopolymeric matrix of the composite, we propose the GP0 formulation optimized in a previous study (Dal Poggetto et al. 2021a). The release mechanism in GP0 and in the cork-based composite geopolymer results in the expulsion from the pores of those of elements trapped in the pores that did not react during the activation reaction. In addition, a dissolution process of soluble species in an alkaline environment such as that of the geopolymers can also be taken into account. The introduction of cork dust reduces or leaves unchanged the release of almost all ions. A possible explanation for this phenomenon is that the cellular structure of cork inhibits the diffusion of ions from the geopolymer pores to the eluate solution.

### Chemical and NMR analyses on eluate

**Fig. 3:**
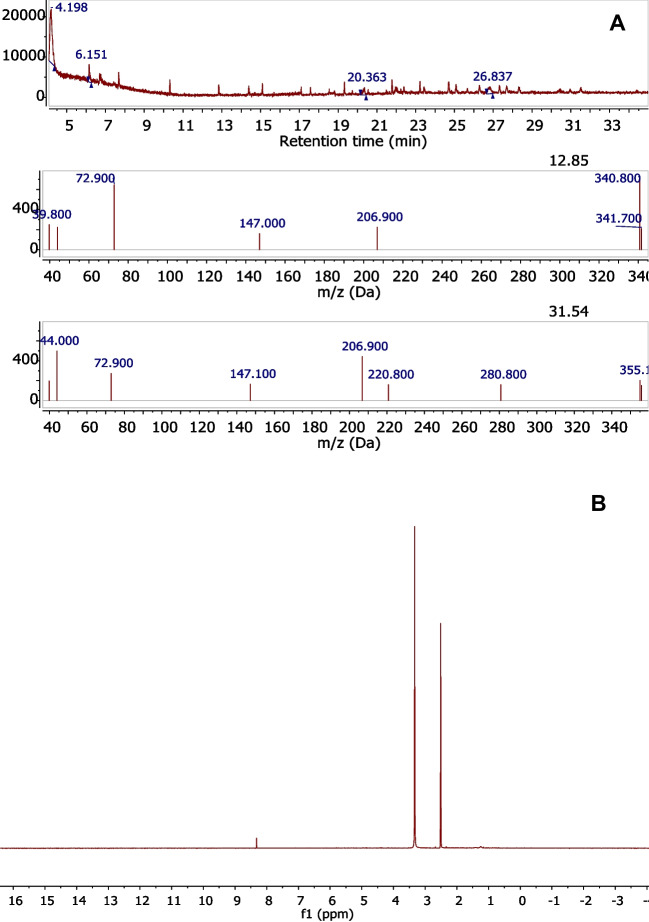
**A** GC–MS: direct injection of 10 μL of solution spectra of leachate solutions and **B** ^1^HNMR: lyophilized sample from 5 mL of solution spectra of leachate solutions

NMR and GC–MS analyses are frequently employed techniques to detect organic molecules extracted from cork materials (Ferreira et al. 2012; Pinto et al. 2019). However, even after repeated runs, both the NMR and the GC–MS analysis (see Fig. 3) failed to record any identifiable signal attributable to molecular species present in the leachate solutions, suggesting a negligible release of low molecular weight organic compounds from the geopolymers. All detectable peaks in the GC–MS spectra were attributed to silane compounds released from the column with the use of Agilent’s MassHunter Software. At trace levels, weak signals compatible with long-chain aliphatic compounds were observed by NMR between 1.5 and 0.5 ppm. This can derive from the depolymerization of suberin, one of the main components of cork, which can be slightly hydrolysed in the alkaline environment necessary for the geopolymer formation (Mission and Cocero 2022) with higher probability in cork not treated for food processing (Weiwei et al. 2019).

### Mineralogical analysis

**Fig. 4:**
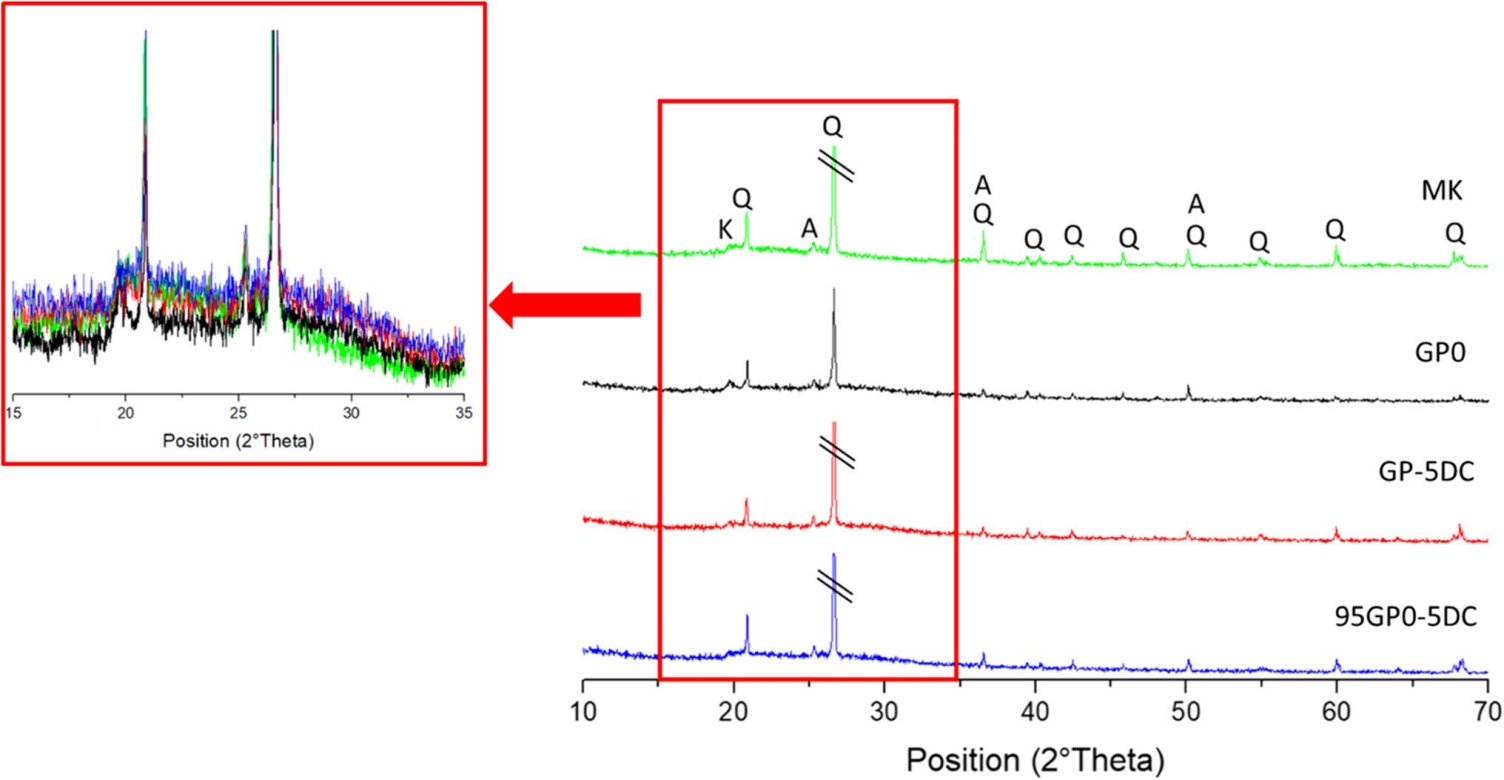
XRD spectra of metakaolin (MK), GP0, GP-5DC, and 95GP0-5DC cured for 28 days

Using the XRD technique, the role of waste cork was investigated to understand whether or not it had altered the reticulation degree of the geopolymer. The geopolymer network is an aluminosilicate network with no long-range order, showing a characteristic amorphous peak slightly shifted towards higher 2θ values with respect to the metakaolin diffuse reflection (around 22–24°), as seen in Fig. 4, enlarged spectra. This peak is also seen in geopolymers with cork added both before and after alkaline activation. In particular, all geopolymers show diffuse reflections typical of an amorphous aluminosilicate network, appearing at about 26–28° in 2θ (Temuujin et al. 2009; Dal Poggetto et al. 2022; Dal Poggetto et al. 2021a).

Figure 4 also shows discernible sharper peaks corresponding to anatase and alpha-quartz, which are virtually identical for all compositions, indicating a remarkable similarity among the samples. This observation convincingly demonstrates that the discarded cork used in this study does not significantly disturb or disrupt the geopolymeric network (Dal Poggetto et al. 2021a).

### Apparent density, real density, and porosity

**Fig. 5:**
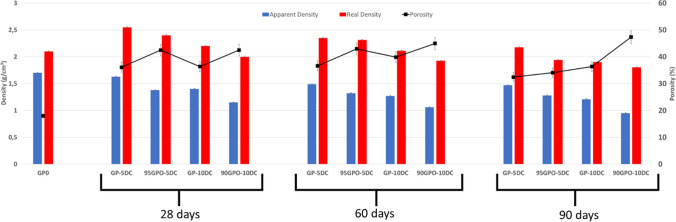
Comparison of density and porosity values as a function of increasing days of ageing

Figure 5 shows the apparent and real densities associated with the porosity of geopolymers containing 5% and 10% of cork dust (before and after alkaline activation) and after 28, 60, and 90 days of ageing. The role of cork dust, a material known to be very light and porous, is evident. J. Ordovás et al. (Ordovás et al. 1996) report that cork tissue is made up of dead cells from 30 to 40 µm in size, with no spaces between them; this means that cork has an internal porosity which is partially blocked. Due to this particular morphology, we observed a decrease in density proportional to the amount of cork added in both series of composites, regardless of when the alkaline activation takes place. However, for the same cork content, the decrease in density is more pronounced when the cork is added after the alkaline activation, probably because in this case the polymerization reaction is already somewhat advanced. By increasing the ageing time from 28 to 60 days, both the apparent and real densities decrease slightly, always proportionally to the amount of cork dust added. At 90 days ageing time, especially for the real density, we see a levelling of the values (except for GP-5DC), as if the material had reached saturation. The samples after alkaline activation show a higher porosity compared to the samples mixed before alkaline activation; both before and after alkaline activation, the porosity increases as more cork is added. In all cases, the porosity of the samples decreases with increasing ageing time.

### Microstructural analysis

**Fig. 6:**
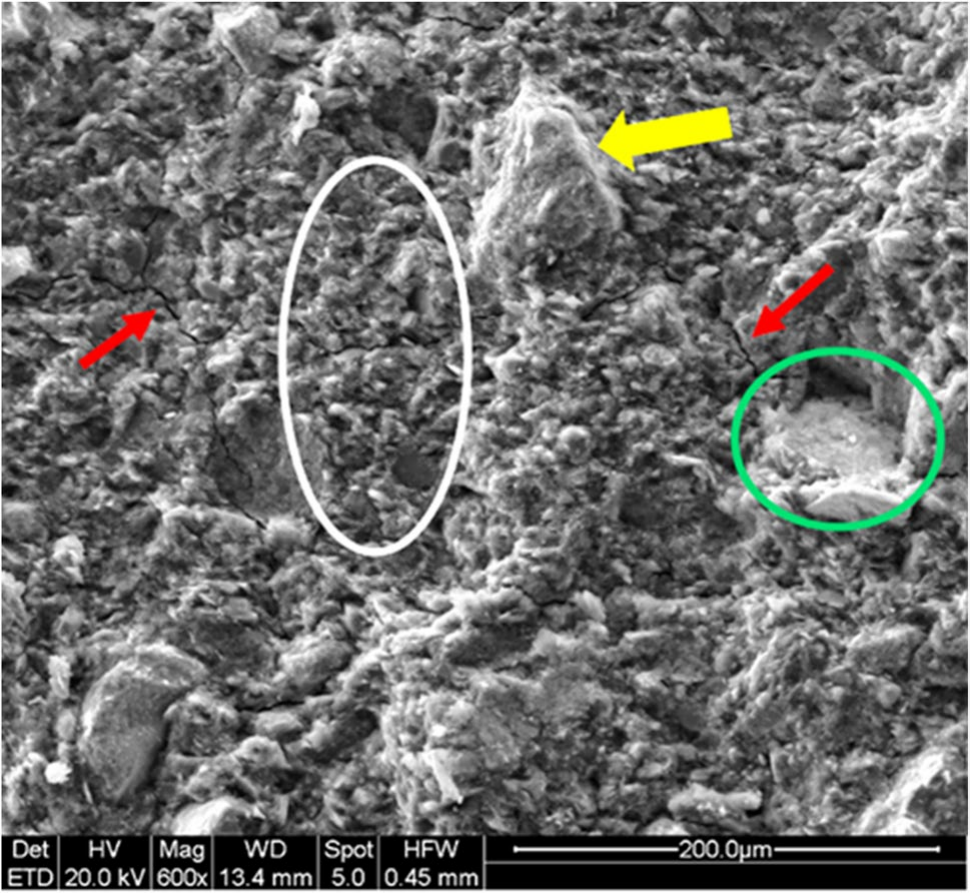
Scanning electron microscopy images taken at low magnification of GP0

**Fig. 7:**
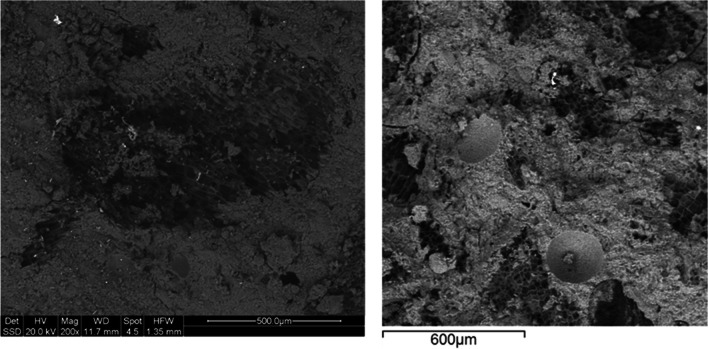
Scanning electron microscopy images taken at low magnification of GP-5DC (left) and 95GP0-5DC (right)

Metakaolin-based geopolymers have been widely investigated (Duxson et al. 2007) due to the high reactivity of MK in alkaline environments. Their microstructures vary with the Si:Al molar ratio (Davidovits 2008; Temuujin et al. 2009), but have a very recognizable morphology as observed by SEM in all the samples described in this paper (Figs. 6 and 7). The platy metakaolin particles were absent, indicating complete dissolution in the alkaline activator solutions. In order to better study the microstructure, we compared the structure of the geopolymer composites with discarded cork to the reference material, GP0. Figure 6 shows an image of GP0, where the dense, characteristic granular structure, indicative of successful geopolymerization, can be seen in the regions in the white circle (Dal Poggetto et al. 2021a; Dal Poggetto et al. 2022; Dal Poggetto et al. 2021b). Yellow arrows indicate unreacted alpha-quartz grains remaining after the reaction of MK with the alkaline solution. Red arrows point to crack deflection, frequently accompanied by pull-outs (green areas), indicating zones where quartz grains from the pristine MK did not react optimally. When pulled out of the mold, these areas immediately form cracks due to the fragility of the incompletely reacted structure.

In Fig. 7, the GP-5DC is displayed on the left, while the 95GP0-5DC is presented on the right. The primary distinction between these formulations, as previously explained, lies in the introduction of cork before and after alkaline activation. In both samples, the presence of cork remains distinctly visible, confirming earlier observations that cork does not interfere with the geopolymerization process. Notably, the 95GP0-5DC sample exhibits visible pores, which are absent in the GP-5DC. Moreover, the 95GP0-5DC displays more pronounced fractures compared to the GP-5DC, aligning with the fragility observed in the mechanical tests (refer to the next section).

### Mechanical properties

**Fig. 8:**
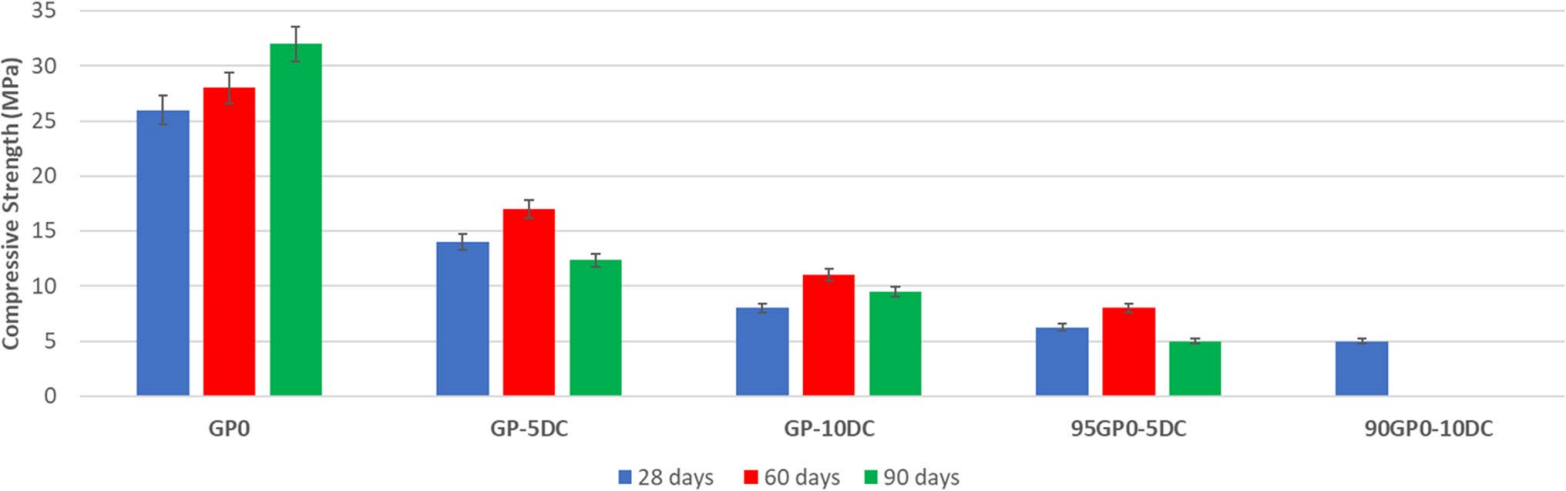
Compressive strength of all sample after 28, 60, and 90 days

Compressive strength tests were performed on all the geopolymer samples after 28, 60, and 90 days of curing (Fig. 8). The properties of GP0 are consistent with the results of previous studies (Davidovits and Davidovics 1988; Duxson et al. 2007). In contrast, the characteristics of the samples containing cork are interesting. After 28 days, these cork-containing samples exhibit significantly lower mechanical properties than GP0, and this trend continues with higher percentages of cork, whether added before or after alkaline activation. At the 60-day and 90-day marks, it is noticeable that the 90GP0-10DC does not give any measurable results. This is not due to the integrity of the geopolymers being compromised but rather the impossibility of testing them. The excessive presence of unreacted cork has caused the 90GP0-10DC to absorb a considerable amount of water, making the sample malleable but resistant to breakage. Regarding the other samples, there is a noticeable peak in their properties at 60 days, followed by a decline at 90 days. The addition of cork after alkaline activation can indeed give the material light and ductile properties. However, if it is to be used as a reinforcement, it must be incorporated with MK before alkaline activation.

### Antibacterial properties

**Fig. 9:**
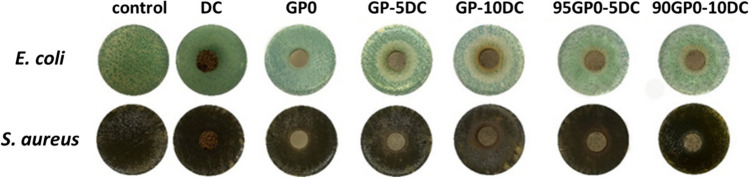
Images of *E. coli* and *S. aureus* plates alone (for the control) and in the presence of the discarded cork (DC), GP0, GP-5DC, GP-10DC, 95GP0-5DC, and 90GP0-10DC

The images of all the geopolymer samples comparing the cork dust and the reference GPO are shown in Fig. 9 after 24 h of incubation with the bacteria. It can be observed that no inhibition zones higher than the disc dimensions can be seen on the *S. aureus* plates. On the other hand, two phenomena can be observed on the plates incubated with *E. coli*. When the discarded cork is mixed with the dry MK before the alkali activation (as in the case of GP-5DC and GP-10DC), two types of zones can be observed: one closer to the sample disc, where no microbial growth is detected, and the larger one, where the microbial growth seems to be reduced. These effects may be due to the combination of the alkaline microenvironment provided by the geopolymer discs and water uptake from the bacterial media, as they are not seen in the DC plate alone. The latter can interfere with the microbial uptake of nutrients from the media, thereby slowing down the growth. When the DC is added after alkali activation (as in the case of 95GP0-5DC and 90GP0-10DC), only the zone of reduced microbial growth can be observed. The latter is undoubtedly due to the DC as it increases from 5 (GP-5DC; 95GP0-5DC) to 10 wt% to (GP-10DC; 90GP0-10DC).

**Fig. 10:**
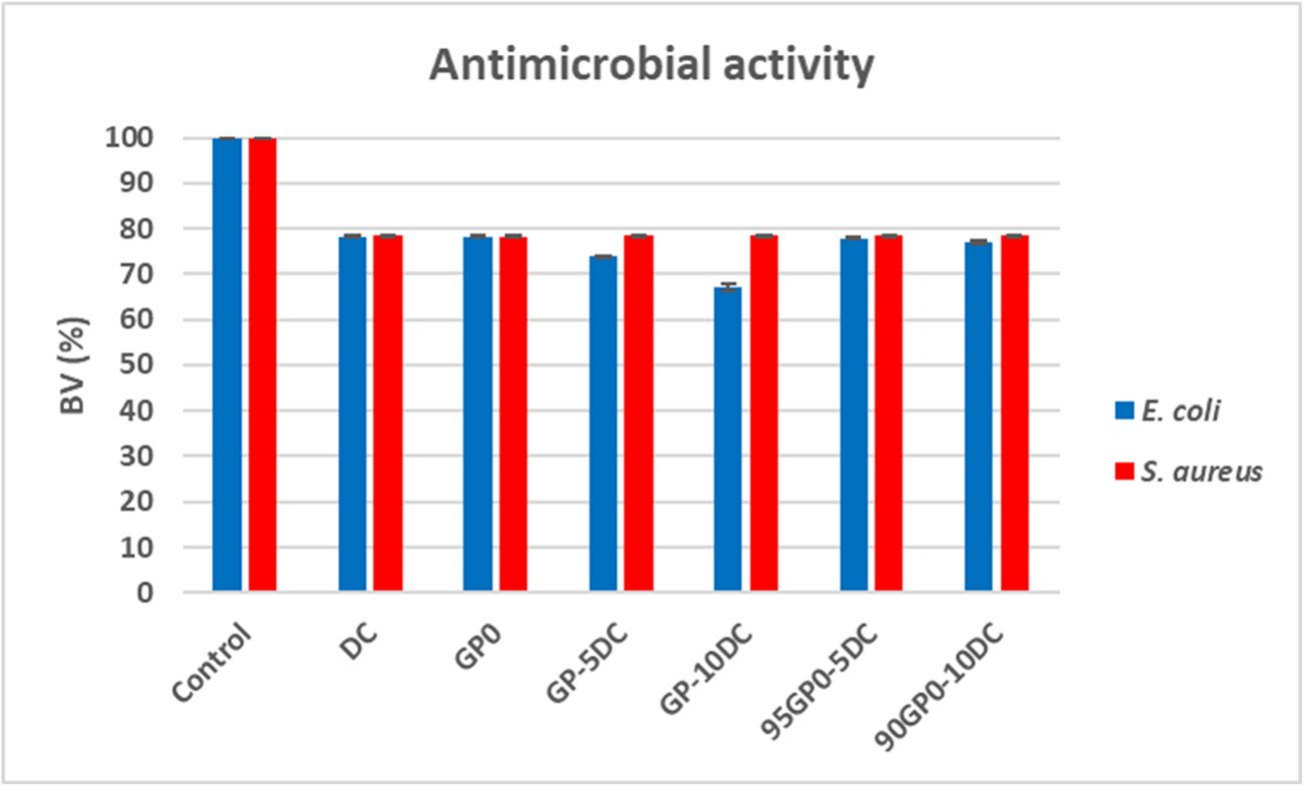
Bacterial viability, BV (%), of the microbial strains after incubation with DC, GP0, GP-5DC, GP-10DC, 95GP0-5GP, and 90GP0-10DC

The data on the reduction in the bacterial viability (BV, %) of the microbial strains compared to the controls (representing the 100% of BV) are shown in Fig. 10. The results show that there is a reduction in the BV from 100 to 78% as no microbial growth was detected on any of the sample discs. Furthermore, the samples with the greater reduction in the BV (72% and 67% for *E. coli*) were those in which the DC was added before the alkaline activation, the case in which the cork comes into contact and adsorbs the alkaline activator solutions. Although the NMR and GC–MS results suggest that there is no release of water-soluble lignin, cellulose, and hemicellulose fractions with antimicrobial activity (Yun et al. 2021), the combination of the unreacted alkaline activator retained in the cork with the culture medium may extract some minor contaminants, as evidenced by the yellowish halo (see Fig. 9) around the geopolymer discs for the four samples with cork dust (GP-5DC, GP-10DC, 95GP0-5DC, and 90GP0-10DC).

## Conclusions

The study provides a comprehensive overview of the interplay between material components, processing conditions, and resulting properties in metakaolin-based geopolymers with cork. The evaluation of metakaolin-based geopolymers with and without cork powder reveals complex interactions between components and their effects on various properties.

- Leaching test results show significant release of aluminum, calcium, silicon, sodium, and potassium. Predominantly aluminum is released from the metakaolin-based geopolymer (10 ppm), while Na presence remains constant between 5 and 7.5 ppm, indicating unaffected geopolymerization.
- NMR and GC–MS analyses suggest negligible release of low molecular weight organic compounds from cork dust.
- XRD spectra consistently show characteristic features of an almost amorphous aluminosilicate structure around 26°, indicating that cork does not significantly disrupt the geopolymer network.
- Density and porosity analyses highlight the role of cork in reducing density, particularly after alkaline activation, with porosity increasing and density decreasing over time, reaching saturation at 90 days.
- SEM microstructural observations confirm that cork does not interfere with geopolymerization, and mechanical properties show that cork provides a trade-off between lightness and ductility, particularly after alkaline activation.
- Antibacterial observations suggest potential inhibitory effects on microbial growth due to alkaline microenvironment and DC presence which remove water from bacterial media reducing nutritive substance intake, providing insights for tailoring cork/geopolymer composites with low environmental impact and favorable performance for specific applications.

In general, the addition of cork dust after alkaline activation provides less benefit in terms of both mechanical and chemical structure than the addition of cork prior to alkaline activation. Despite variations in porosity over time, the samples remain crush resistant and may be suitable for the construction of durable, moisture resistant interior panels.

## Data Availability

The data are available on request.

## References

[CR1] Adhikary SK, D’Angelo A, Viola V, Catauro M, Perumal P (2024). Alternative construction materials from industrial side streams: are they safe?. Energ Ecol Environ.

[CR2] Bajpai R, Choudhary K, Srivastava A, Sangwan KS, Singh M (2020). Environmental impact assessment of fly ash and silica fume based geopolymer concrete. J Clean Prod.

[CR3] Bakhtyar B, Kacemi T, Nawaz MA (2017). A review on carbon emissions in Malaysian cement industry. Int J Energy Econ Policy.

[CR4] Baltazar LG, Henriques FMA, Temporão D, Cidade MT (2019). Experimental assessment of geopolymer grouts for stone masonry strengthening. Key Eng Mater.

[CR5] Boldrini G, Sgarlata C, Lancellotti I, Barbieri L, Giorgetti M, Ciabocco M, Zamponi S, Berrettoni M, Leonelli C (2021). Efficient chemical stabilization of tannery wastewater pollutants in a single step process: geopolymerization. Sustain Environ Res.

[CR6] Catauro M, D’Errico Y, D’Angelo A, Clarke RJ, Blanco I (2021). Antibacterial activity and iron release of organic-inorganic hybrid biomaterials synthesized via the sol-gel route. Appl Sci.

[CR7] Catauro M, D’Angelo A, Fiorentino M, Pacifico S, Latini A, Brutti S, Vecchio Ciprioti S (2023). Thermal, spectroscopic characterization and evaluation of antibacterial and cytotoxicity properties of quercetin-PEG-silica hybrid materials. Ceram Int.

[CR8] Chang Z-T, Song X-J, Munn R, Marosszeky M (2005). Using limestone aggregates and different cements for enhancing resistance of concrete to sulphuric acid attack. Cem Concr Res.

[CR9] Chen S, Wu C, Yan D (2019). Binder-scale creep behavior of metakaolin-based geopolymer. Cem Concr Res.

[CR10] Chen S, Wu C, Yan D, Ao Y, Ruan S, Zheng W, Sun X, Lin H (2021). Relation between drying shrinkage behavior and the microstructure of metakaolin-based geopolymer. J Zhejiang Univ A.

[CR11] Clausi M, Tarantino SC, Magnani LL, Riccardi MP, Tedeschi C, Zema M (2016). Metakaolin as a precursor of materials for applications in cultural heritage: geopolymer-based mortars with ornamental stone aggregates. Appl Clay Sci.

[CR12] D’Angelo A, Dal Poggetto G, Piccolella S, Leonelli C, Catauro M (2022). Characterisation of white metakaolin-based geopolymers doped with synthetic organic dyes. Polymers.

[CR13] D’Angelo A, Vertuccio L, Leonelli C, Alzeer MIM, Catauro M (2023). Entrapment of acridine orange in metakaolin-based geopolymer: a feasibility study. Polymers.

[CR14] Dal Poggetto G, Catauro M, Crescente G, Leonelli C (2021). Efficient addition of waste glass in MK-based geopolymers: microstructure, antibacterial and cytotoxicity investigation. Polymers.

[CR15] Dal Poggetto G, D’Angelo A, Blanco I, Piccolella S, Leonelli C, Catauro M (2021). FT-IR study, thermal analysis, and evaluation of the antibacterial activity of a MK-geopolymer mortar using glass waste as fine aggregate. Polymers.

[CR16] Dal Poggetto G, D’Angelo A, Catauro M, Barbieri L, Leonelli C (2022). Recycling of waste corundum abrasive powder in Mk-based geopolymers. Polymers.

[CR17] Davidovits J, Davidovics M (1998) Alkaline aluminosilicate geopolymeric matrix for composite materials with fiber reinforcement and method for obtaining same. US patent 5: 798, 307. EP0815064B1 1999-09-01

[CR18] Davidovits J, Davidovics M (1988). Geopolymer: room temperature ceramic matrix for composites. Ceram Eng Sci Proc.

[CR19] Davidovits J (2008) Geopolymer, chemistry and applications, (3rd printing), Institut Geopolymer, Saint-Quentin, France 585

[CR20] Dimas D, Giannopoulou I, Panias D (2009). Polymerization in sodium silicate solutions: a fundamental process in geopolymerization technology. J Mater Sci.

[CR21] Duxson P, Fernández-Jiménez A, Provis JL, Lukey GC, Palomo A, van Deventer JSJ (2007). Geopolymer technology: the current state of the art. J Mater Sci.

[CR22] EN 197–5 (2021) Cement - Part 5: Portland-composite cement CEM II/C-M and composite cement CEM VI. https://standards.iteh.ai/catalog/standards/cen/69d3b559-4114-43b3-bfed-05787fc839a2/en-197-5-2021. Accessed 2 Jan 2024

[CR23] EN 197–6 (2023) Cement - Part 6: Cement with recycled building materials. https://standards.iteh.ai/catalog/standards/cen/96772c60-54f9-4226-b230-8f5c39dbd57d/en-197-6-2023. Accessed 2 Jan 2024

[CR24] Ferreira R, Garcia H, Sousa AF, Petkovic M, Lamosa P, Freire CSR, Silvestre AJD, Rebelo LPN, Pereira CS (2012). Suberin isolation from cork using ionic liquids: characterisation of ensuing products. New J Chem.

[CR25] Geraldes CFM, Lima AM, Delgado-Rodrigues J, Mimoso JM, Pereira SRM (2016). Geopolymers as potential repair material in tiles conservation. Appl Phys A.

[CR26] Hadjsadok A, Kenai S, Courard L, Michel F, Khatib J (2012). Durability of mortar and concretes containing slag with low hydraulic activity. Cement Concr Compos.

[CR27] Haga K, Sutou S, Hironaga M, Tanaka S, Nagasaki S (2005). Effects of porosity on leaching of Ca from hardened ordinary Portland cement paste. Cem Concr Res.

[CR28] Hudzicki J (2009) Kirby-Bauer disk diffusion susceptibility test protocol. Am Soc Microbiol 15:55–63. https://asm.org/getattachment/2594ce26-bd44-47f6-8287-0657aa9185ad/Kirby-Bauer-Disk-DiffusionSusceptibility-Test-Protocol-pdf.pdf. Accessed 2 Jan 2024

[CR29] Longhi MA, Rodríguez ED, Walkley B, Zhang Z, Kirchheim AP (2020). Metakaolin-based geopolymers: relation between formulation, physicochemical properties and efflorescence formation. Compos B Eng.

[CR30] Ma Y, Hu J, Ye G (2013). The pore structure and permeability of alkali activated fly ash. Fuel.

[CR31] Malchiodi B, Marchetti R, Barbieri L, Pozzi P (2022). Recovery of cork manufacturing waste within mortar and polyurethane: feasibility of use and physical, mechanical, thermal insulating properties of the final green composite construction materials. Appl Sci.

[CR32] Mission EG, Cocero MJ (2022). Accessing suberin from cork via ultrafast supercritical hydrolysis. Green Chem.

[CR33] Moutinho S, Costa C, Andrejkovičová S, Mariz L, Sequeira C, Terroso D, Rocha F, Velosa A (2020). Assessment of properties of metakaolin-based geopolymers applied in the conservation of tile facades. Constr Build Mater.

[CR34] Nair DG, Fraaij A, Klaassen AAK, Kentgens APM (2008). A structural investigation relating to the pozzolanic activity of rice husk ashes. Cement Concrete Res.

[CR35] Novais RM, Saeli M, Caetano APF, Seabra MP, Labrincha JA, Surendran KP, Pullar RC (2019). Pyrolysed cork-geopolymer composites: a novel and sustainable EMI shielding building material. Constr Build Mater.

[CR36] Ordovás J, Carmona E, Moreno MT, Ortega MC (1996). Characteristics of internal porosity of cork container media. Hortoscience.

[CR37] Pinto J, Oliveira AS, Lopes P, Roseira I, Cabral M, Bastos MdL, Guedes de Pinho P (2019). Characterization of chemical compounds susceptible to be extracted from cork by the wine using GC-MS and ^1^H NMR metabolomic approaches. Food Chem.

[CR38] Provis JL, Ismail I, Myers RJ, Rose V, Van Deventer JSJ (2011). Characterising the structure and permeability of alkali-activated binders. Int RILEM Conf Adv Constr Mater through Sci Eng.

[CR39] Righi C, Barbieri F, Sgarbi E, Maistrello L, Bertacchini A, Andreola FN, D’Angelo A, Catauro M, Barbieri L (2022). Suitability of porous inorganic materials from industrial residues and bioproducts for use in horticulture: a multidisciplinary approach. Appl Sci.

[CR40] Rovnaník P (2010). Effect of curing temperature on the development of hard structure of metakaolin-based geopolymer. Constr Build Mater.

[CR41] Samuel DM, Inumerable N, Stumpf A, Kriven WM (2023). Thermal conductivity of several geopolymer composites and discussion of their formulation. Int J Appl Ceram Technol.

[CR42] Sudagar A, Andrejkovicova S, Patinha C, Velosa A, McAdam A, Ferreira da Silva E, Rocha F (2018). A novel study on the influence of cork waste residue on metakaolin-zeolite based geopolymers. Appl Clay Sci.

[CR43] Temuujin J, Minjigmaa A, Rickard W, Lee M, Williams I, Riessen AV (2009). Preparation of metakaolin based geopolymer coatings on metal substrates as thermal barriers. Appl Clay Sci.

[CR44] Weiwei S, Qingguo W, Yuan Yidan Y, Xiaozhou S, Mingqiang Z (2019). Alkaline solvent cooking treatment of cork and component analysis of filtrates. Wood Res.

[CR45] Ye N, Yang J, Liang S, Hu Y, Hu J, Xiao B, Huang Q (2016). Synthesis and strength optimization of one-part geopolymer based on red mud. Constr Build Mater.

[CR46] Yun J, Wei L, Li W, Gong D, Qin H, Feng X, Li G, Ling Z, Wang P, Yin B (2021). Isolating high antimicrobial ability lignin from bamboo kraft lignin by organosolv fractionation. Front Bioeng Biotechnol.

[CR47] Zaid O, Martínez-García R, Abadel AA, Fraile-Fernández FJ, Alshaikh IMH, Palencia-Coto C (2022). To determine the performance of metakaolin-based fiber-reinforced geopolymer concrete with recycled aggregates. Arch Civ Mech Eng.

[CR48] Zhang Z, Yao X, Zhu H (2010). Potential application of geopolymers as protection coatings for marine concrete I. Basic properties. Appl Clay Sci.

